# Commentary: Coronary stent deformation induced by guide catheter extension

**DOI:** 10.3389/fcvm.2024.1357337

**Published:** 2024-03-06

**Authors:** Sotirios C. Kotoulas, Andreas S. Kalogeropoulos, Pim A. L. Tonino, Andreas S. Triantafyllis

**Affiliations:** ^1^ Department of Cardiology, Asklepeion General Hospital, Athens, Greece; ^2^ Department of Cardiology, Mitera General Hospital, Hygeia HealthCare Group, Athens, Greece; ^3^ Department of Cardiology, Catharina Ziekenhuis Eindhoven, Eindhoven, Netherlands

**Keywords:** Guidezilla™, complex coronary lesions, transradial, guide extension catheters, tips and tricks, coronary stent deformation

A Commentary on Guidezilla™ guide extension catheter I for transradial coronary intervention By Lei XJ, Liang Q, Fang Y, Xiao YH, Wang DQ, Dong MZ, Li JC, Yu T (2022). Front. Cardiovasc. Med. 9:931373. doi: 10.3389/fcvm.2022.931373

We read with great interest the elegantly written article by Lei et al. regarding the use of the Guidezilla I (Boston Scientific) guide catheter extension (GCE) during transradial percutaneous coronary intervention (PCI) (1). The authors report excellent efficacy and safety in the utilization of GCE during PCI, irrespective of the SYNTAX score (1). The authors mention shaft breakage, stent stripping or dislodgement, coronary artery dissection/perforation, and entrapment as possible complications when using GCEs (1). Even though GCEs facilitate the delivery of intracoronary material (stents, balloons), in complex coronary anatomies (calcification, tortuosity, presence of previous stents) stent damage induced by GCE has been reported (2–4). We comment on the underlying mechanisms of stent deformation during its insertion in a GCE while we propose helpful tips to avoid this complication. The frequent use of GCEs in challenging PCIs, mandates for early recognition and timely intervention of possible complications related to GCEs.

We have noted, in our clinical practice, stent deformation at the entry port of GCEs. Malalignment between the stent and the collar at the entry port of the GCE is the most common cause for encountering resistance to advancement, which can result in stent deformation, if aggressive push persists (3). It is noteworthy that positioning the entry port of the GCE in an arterial bend (anonymous artery for the radial and aortic arch for the femoral approach) may increase friction between the interventional material and the collar (4). The use of a smaller diameter GCE (6-Fr) in a larger diameter GC (7-Fr), may result in an inter-catheter diameter gap which can aggravate malalignment (4). Additionally, the use of two guidewires inside the GCE or twisting between the guidewire and the pushrod may hamper smooth insertion of the stent in the entry port of the GCE. Loading the stent on the guidewire and GCE on the table, outside the patients' body, has been described, as a bailout technique (4). Moreover, newer generation devices are available in larger diameters ensuring improved deliverability and alignment. The Guidezilla II GCE (Boston Scientific) claims to provide self-alignment upon insertion into the GC just by keeping the hub look upwards (Z letter facing up). Furthermore, the incorporation of the novel hydrophilic coating, coupled with the redesigned hub, facilitates smoother delivery, while the shorter hypotube transition length minimizes the device interaction between the stent and the GCE. The Guideliner V3 GCE (Teleflex) claims to minimize device/collar interaction by aligning devices through a half-pipe transition channel. The Telescope (Medtronic) GCE has a half pipe technology designed to minimize device/collar interaction allowing seamless delivery (5–7). Differences between various types of GCEs are illustrated in Table 1.

**Table 1:** Comparison of GCEs: Guidezilla 1 vs. Guidezilla 2 vs. Guideliner V3 vs. Telescope.

	Guidezilla I	Guidezilla II	Guideliner V3	Telescope
Available sizes	6F	6F, 7F, 8F	5F, 6F, 7F, 8F	6F, 7F
Guide segment	25 cm	40 cm on 6F Long	25 cm	25 cm
Special features	Hub design without marker	Z marker: Augments self-alignment into the Guide Catheter	Half – Pipe Transition channel: Minimizes device/collar interaction by aligning devices	Half pipe technology designed to minimize device/collar interaction allowing seamless delivery

In conclusion, stent deformation during insertion in a GCE is an infrequent complication. Good alignment between the stent and GCE, 1:1 diameter compatibility, avoidance of locating the transition collar in arterial bends, the use of a single wire inside the GCE, keeping the guidewire and pushrod separated or even loading the stent in the GCE outside the patients' body, and the use of newer generation devices are essential measures to prevent this complication.
